# King Edward's Hospital Fund for London

**Published:** 1912-04-27

**Authors:** 


### KING EDWARD'S HOSPITAL FUND FOR LONDON.

The annual meeting of the Governors and General Council of King Edward's Hospital Fund for London, to receive the accounts and the report of the General Council for the year 1911, was held at St. James's Palace on the 18th inst., the Speaker of the House of Commons being in the chair.

Those present were: The Viscount Iveagh, the Earl of Bessborough, Lord Rothschild, the President of the Hospital Sunday and Hospital Saturday Funds (the Right Hon. the Lord Mayor), the President of the Royal College of Physicians (Sir Thomas Barlow, Bart.), the President of the Royal College of Surgeons (Mr. Rickman J. Godlee), the Right Hon. Sir Joseph Dimsdale, Bart., the Right Hon. Sir Savile Crossley, Bart. (Hon. Secretary), the Right Hon. Thomas Burt, M.P., the Right Hon. Sir Edgar Speyer, Bart., Sir W. S. Church, Bart., Sir Horace Marshall, Sir William J. Collins, Sir John Craggs, Mr. Frederick M. Fry, Dr. Edwin Freshfield, Mr. John G. Griffiths (Hon. Secretary).

The minutes of the last meeting having been read and confirmed, letters of regret were reported from the following: H.H. the Duke of Teck, the Duke of Norfolk, the Bishop of London, the Bishop of Southwark, Cardinal Bourne, the Rev. T. Bowman Stephenson, D.D., Lord Revelstoke, Lord Farquhar, Lord Stamfordham, the Right Hon. the Master of Elibank, M.P., the Postmaster-General (the Right Hon. Herbert Samuel, M.P.), the President of the Local Government Board (the Right Hon. John Burns, M.P.), the Governor of the Bank of England (Mr. A. C. Cole), the Right Hon. Sir Ernest Cassel, Sir Trevor Lawrence, Bart., Sir Julius Wernher, Bart., Professor Sir William Osler, Bart., Sir Henry Burdett, Mr. J. Danvers Power, Rev. Canon Barnett, Mr. William Latham, K.C., Mr. J. Pierpont Morgan, jun., Mr. Albert G. Sandeman.

Lord Rothschild (the Honorary Treasurer), in presenting the account of receipts and expenditure, and the balance-sheet for the year ending December 31, 1911, said he was glad to announce that there was a small increase in the capital account, but it was much to the regret of the Council that more financial support and sympathy were not forthcoming for the Fund, as, owing to recent legislation, there might be increased calls upon the various hospitals. When the status of King Edward's Hospital Fund for London was definitely arranged by Parliament, the Treasurer and Finance Committee were ordered to separate their investments into trust securities and non-trust securities. Any money given to the Fund without an expression of preference by the donor would naturally be invested in trust securities. It was always left to the donor to express a wish that his contribution should be invested in non-trust securities. The result of that legislation had been as follows: The total original value of the Fund's present investments was £1,670,864, of which £608,438 represented trust securities and £1,062,426 non-trust securities. The present value of the trust securities was £574,375, or a diminution of 5.6 per cent.; of the non-trust securities £1,119,629, being an increase of 5.4 per cent.; making a total of £1,694,004, showing a net increase of 1.4 per cent. He trusted that when he had the honour to move the adoption of the accounts in another year he would be able to announce an increase of contributions.

The Speaker of the House of Commons formally moved the adoption of the accounts.

The resolution, having been seconded by Lord Iveagh, was then put and carried unanimously.

Sir Savile Crossley (Honorary Secretary) presented the draft report of the Council for the year 1911, as follows :—

### REPORT OF THE GENERAL COUNCIL

*For the Year ending December 31, 1911.*

*Receipts.*

The total receipts for the year 1911 were £235,620 9s. 9d. This sum was made up as follows: Donations, £48,106 18s. 4d.; contributions to capital, £6,071 10s. 1d., including £1,239 17s. 7d. on account of a legacy, together with £1,444 12s. 3d. net profit on realisation of securities; annual subscriptions, £25,766 2s. 9d.; contribution of the League of Mercy, £17,000; from the Lewis Estate, £13,491 5s. 11d.; legacies (other than legacies to capital), £48,576 16s. 6d.; interest from investments, £76,407 16s. 2d.; together with £200 from the trustees of the Bawden Fund.

The donations include a munificent contribution of £31,500 made to the Fund by Sir Ernest Cassel, being part of a gift of £50,000 to Hospitals in London and the provinces, in memory of his daughter, the late Mrs. Maude Ashley. The Governors and the General Council are deeply grateful to Sir Ernest Cassel for entrusting the Fund with the distribution of this large sum and thus enabling the awards which had already been voted to hospitals and other institutions to be increased in each instance by one-fifth.

*Grants to Hospitals, Convalescent Homes, and Sanatoria.*

The amount originally placed at the disposal of the Distribution and Convalescent Homes Committees for distribution was £157,500, being an increase of £2,500 over the sum distributed in 1910. The supplementary grants made in respect of the special donation above referred to increased the total distribution to £189,000. Of this the hospitals received £181,200, while the remaining £7,800 was distributed among consumption sanatoria and convalescent homes taking London patients. The total sum distributed among hospitals, convalescent homes, and consumption sanatoria during the last ten years (after deducting grants to the extent of £7,409 16s. 7d. that have been allowed to lapse) is £1,243,840 3s. 5d.; since the foundation of the Fund a total amount of £1,475,916 8s. 5d. has been distributed.

*Expenses of Administration.*

During the year the amount spent on administration (including the expenses of the Out-patient Inquiry Committee) was £1 9s. 11d. per cent. of the total amount received. The corresponding percentage for the whole period since the inauguration of the Fund has been £1 5s. 3d.

*Legacies, Donations, and Subscriptions.*

Among the legacies received during the year were payments of £28,301 12s. 1d. on account of the residue of the estate of the late Mr. Edward Matyear, £14,643 5s. 11d. on account of the residue of the estate of the late Mr. G. H. Swonnell, and £2,936 4s. 7d. from the late Mr. B. C. Hirsch. The Council have also to acknowledge an anonymous donation of £10,000, and an annual subscription of £5,000 from Mr. Walter Morrison. His Majesty the King was graciously pleased to allocate to the Fund a sum of £2,000 out of the Coronation gift of His Highness the Maharajah of Gwalior. The amounts received in donations and subscriptions, excluding gifts to capital, show, as compared with 1910, an increase of £28,376 12s. 2d.

*League of Mercy.*

The League of Mercy has this year contributed £17,000. During the twelve years of its existence the League has furnished in all £170,000 to the Fund. The Council express their best thanks to the voluntary workers who have so materially assisted the Fund by the collection of this large sum.

*Work of the Fund's Visitors.*

Every eligible hospital and consumption sanatorium which applied for a grant was inspected and reported upon by the Fund's Visitors, consisting as heretofore of one medical and one lay man in each case.

*Beds at Consumption Sanatoria.*

The scheme of the Convalescent Homes Committee for securing beds at consumption sanatoria and placing them at the disposal of London hospitals for the accommodation of patients urgently needing country treatment, has proved successful from the point of view both of the sanatoria and of the hospitals. The scheme has this year been extended, the number of beds secured for the coming year amounting to thirty-five at four sanatoria, as against eighteen at three sanatoria last year.

*National Insurance Act.*

In July 1911 the Executive Committee had under consideration the probable effect of the National Insurance Bill upon the future of the hospitals of London, and a subcommittee was appointed to collect information on the subject. A report of this sub-committee, summarising the opinions of thirty-two of the principal hospitals upon various aspects of the question, was published in the newspapers of July 19, 1911.

*Reductions in Cost at London Hospitals.*

The annual statistical report on the expenditure of London hospitals has again been enlarged. It now deals with 106 hospitals, and forms as nearly as possible a complete analysis of the expenditure of all the hospitals which have adopted the Revised Uniform System of Accounts. The reductions in cost recorded in the successive issues of these reports since the comparative figures were first prepared in 1903 now amounts to a total of no less than £47,000 a year.

*Out-patient Inquiry.*

The Special Committee, consisting of Lord Mersey (Chairman), the late Lord Northcote, and the Bishop of Stepney, appointed to inquire into the methods prevailing in the London Voluntary Hospitals with regard to the admission of out-patients, began to sit early in the year. Their proceedings were delayed by the lamented illness and death of Lord Northcote, and the Committee subsequently postponed consideration of their report until the conclusion of the discussion of the National Insurance Bill. The work of the Committee had, therefore, not been completed when the Council for 1911 laid down its duties.

*Hospital Extension or Reconstruction.*

The Distribution Committee have carefully considered all schemes for the extension or reconstruction of hospitals which have been submitted in accordance with the request of the Council. The Fund has thus used its best endeavours to assist the hospital authorities in ensuring that when capital expenditure of this kind is undertaken, adequate consideration is given to economy, efficiency, and the prospect of funds for future maintenance, and has particularly considered the relation of each scheme to the general question of hospital accommodation in London.

*Coronation Durbar.*

In view of the absence from England of His Highness the Duke of Teck and Viscount Iveagh, in connection with the Coronation Durbar in India, the Earl of Donoughmore and the Right Hon. Sir T. Vezey Strong were appointed, in accordance with the provisions of the Act of Incorporation of the Fund, to act with the Speaker as a Committee to exercise the powers of the Governors till their return.

*The Council's Loss.*

The Council regret to record the loss by death, during the year 1911, of two of their earliest members, the Rev. J. Guinness Rogers, D.D., and the late Chief Rabbi, the Rev. Dr. Hermann Adler.

*Retirement of Mr. F. M. Fry.*

The Council desire to express their appreciation of the great services rendered to the Fund by Mr. Frederick M. Fry as Honorary Secretary for the four years ending December 1911. This term included the period immediately following the reorganisation after the Incorporation of the Fund by Act of Parliament, and in this, as well as in other matters, his experience in administrative work and his devotion to the duties of his office have been of special value. The Council are glad to know that the Fund will still retain Mr. Fry's services as a member of the Council and Distribution Committee, as well as in the work connected with the Out-patient Inquiry. The vacancy created by his retirement has been filled by the appointment of Mr. John G. Griffiths, who has been actively associated with the Fund for many years.

*Auditors.*

The President of the Local Government Board, in pursuance of his powers under the Act, has appointed Messrs. Deloitte, Plender, Griffiths and Co. to audit the accounts for the Fund for the year.

*Thanks to the Press.*

The Council are obliged to the Press for the services they have rendered in the past, and continue to rely upon their kindly co-operation in obtaining for the Fund wider publicity and support.

St. James's Palace, April 18, 1912.

The Speaker of the House of Commons, in moving the adoption of the report, said :—

My Lords and Gentlemen,—In accordance with the custom at this annual meeting I intend only to refer briefly to one or two of the matters arising out of the report and accounts which are before us.

A Munificent Donation.

At the distribution meeting in December it was announced that a donor, who was about to distribute an "in memoriam" gift of £50,000 amongst hospitals, had offered the Fund, out of this sum, a donation of £31,500, being the amount required to increase the awards for the year by one-fifth. The donor did not then wish his name to be made known. It was impossible therefore to acknowledge his generosity as we should have wished to do; but I desire now, and I am sure that I shall do it in your name as well as that of the Governors, to tender to Sir Ernest Cassel the best thanks of all connected with the Fund for this most munificent donation—(hear, hear)—and for this mark of his confidence in the principles upon which the distribution of the Fund's grants is made.

The Committee which, under the chairmanship of Lord Mersey, was appointed to inquire into the out-patient system of London has now made considerable progress with the study of the voluminous evidence it has collected, and we look forward to the issue of its report on this difficult subject at no very distant date.

Since the end of the year the Fund has had to mourn the loss of the Duke of Fife, who was one of the oldest members of the Council, and presided over its meetings on several occasions. A resolution was passed by the Executive Committee at the time, expressing in the name of the Governors and the General Council their condolence with their Majesties and the Princess Royal on the loss they had sustained.

I would also desire to refer here to the death of Lord Lister, the most distinguished surgeon of his day, who was for several years a member of the Council of the Fund, a member of the Visiting Committee, and chairman also of the Distribution Committee. We have therefore personal reasons for associating ourselves with the universal tributes to the memory of the noble work of this great man.

Changes in the Personnel of the Fund.

There have been a few changes in the personnel of the Fund since the meeting in December. The Governors have appointed the Rev. R. F. Horton to the Council in the place of the late Rev. J. Guinness Rogers. Mr. Danvers Power is, we regret to say, unable to continue his services on the Distribution Committee, and we have accordingly appointed Mr. Griffiths to that Committee and Mr. H. L. Hopkinson to the Executive Committee.

I now beg to move the adoption of the report, and in doing so I would desire to express the thanks of the Governors and all of us to Lord Rothschild and the members of the Finance Committee for the skill and care with which they watch over our investments. (Hear, hear.)

Sir Edgar Speyer seconded the motion, which was carried unanimously.

On the motion of the Lord Mayor (Sir Thos. Boor Crosby), seconded by Mr. Thomas Burt, a vote of thanks to the Speaker for presiding was carried unanimously.

The Speaker having briefly acknowledged the vote of thanks, the proceedings terminated.

